# Efficacy of Morning Shorter Wavelength Lighting in the Visible (Blue) Range and Broad-Spectrum or Blue-Enriched Bright White Light in Regulating Sleep, Mood, and Fatigue in Traumatic Brain Injury: A Systematic Review

**DOI:** 10.3390/clockssleep6020018

**Published:** 2024-05-28

**Authors:** Chin Moi Chow, Kanchana Ekanayake, Daniel Hackett

**Affiliations:** 1 Sydney School of Health Sciences, Faculty of Medicine and Health, University of Sydney, Sydney 2006, Australia; kanchana.ekanayake@sydney.edu.au (K.E.); daniel.hackett@sydney.edu.au (D.H.); 2 Sleep Research Group, Charles Perkins Centre, University of Sydney, Sydney 2006, Australia

**Keywords:** light therapy, circadian disruption, phase shifts, sleep disturbances, TBI severity

## Abstract

Traumatic brain injury (TBI) profoundly affects sleep, mood, and fatigue, impeding daily functioning and recovery. This systematic review evaluates the efficacy of morning shorter wavelength lighting in the visible (blue) range and broad-spectrum or blue-enriched bright white light exposure in mitigating these challenges among TBI patients. Through electronic database searches up to May 2023, studies assessing sleep, circadian rhythm, sleepiness, mood, and fatigue outcomes in TBI patients exposed to morning shorter wavelength lighting in the visible (blue) range and broad-spectrum or blue-enriched bright white light were identified. Seven studies involving 309 participants met the inclusion criteria. Results indicated consistent advancement in sleep timing among individuals with mild TBI, alongside improvements in total sleep time, mood, and reduced sleepiness with both types of light exposure, particularly in mild TBI cases. Notably, two studies demonstrated alleviation of fatigue exclusively in severe TBI cases following light exposure. Despite promising findings, evidence remains limited, emphasizing the need for future research with standardized protocols to confirm the potential and optimize the benefits of light therapy for TBI recovery.

## 1. Introduction

In Australia, an estimated annual incidence of traumatic brain injury (TBI) ranging from 190,000 to 200,000 cases is extrapolated from New Zealand data [1]. TBIs stem from various causes including sports-related activities, road accidents, workplace incidents, falls, or interpersonal violence [2]. Regardless of severity, TBIs significantly impact lifelong health, affecting work, leisure activities, and relationships. Severe cases often necessitate ongoing care for individuals in coma, vegetative state, or emerging from unconsciousness. Even mild TBI (mTBI) can lead to confusion, disorientation, memory impairment, and various physical symptoms, with fatigue being a prevalent complaint in 53% of individuals [3]. Despite the “mild” label, mTBI’s impact is substantial, constituting 70–90% of all TBI cases [2], with many cases going untreated, complicating incidence accuracy [4].

Sleep and wake disturbances emerge as persistent and disabling sequelae of TBI [5]. Athletes experiencing at least one concussion report poor sleep [6], while 36% of mTBI patients exhibit circadian disruption [7]. Acute sleep issues predict subsequent symptoms like irritability, depression, and headaches in mTBI cases. These disturbances may persist for over 20 years post-injury, affecting a significant proportion of moderate–severe TBI (m-sTBI) patients [8,9]. Various sleep disorders including sleep apnea, post-traumatic hypersomnia, narcolepsy, and periodic limb movements are also reported [10].

Such sleep problems in TBI patients arise from internal and external factors. Damage to brain regions regulating sleep–wake cycles, such as the retinohypothalamic tract and brainstem, contributes to disruptions. Immune responses triggered by injuries may further lead to circadian dysregulation, suggesting bidirectional communication between circadian physiology and immune function [11]. In addressing these challenges, light exposure has been explored as a potential intervention for TBI patients. Recent studies reviewed indicate significant effects of short wavelength lighting range (blue) or blue-enriched white light on reducing depression [12,13,14,15] and fatigue [12,14,15]. Other studies [16,17] extend the investigation to explore the impact of short wavelength lighting (blue) on phase shifts, considering its potent influence on sleep–wake rhythms and associated mechanisms.

Understanding the crucial role of light in human health, particularly its impact on circadian rhythms, is vital. Broad-spectrum white light with all visible wavelengths of 400-700 nm (polychromatic) can reset the circadian clock. However, short wavelength monochromatic light (blue) in the short wavelength range of 446–477 nm with a peak sensitivity at 479 nm in humans), is the strongest synchronizing agent for the clock in humans [18]. Lighting at 570 nm (amber) and at 650 nm (red) are longer wavelengths of light in the visible range. Light entrains the circadian system via the eye, received by intrinsically photosensitive retinal ganglion cells (ipRGC) containing the receptor melanopsin [19]. The melanopsin G family coupled receptor plays an important role in non-image-forming visual functions: entrainment of circadian rhythms, cognitive and affective processes, and hormone secretion [20]. Notably, cones and rods may participate in these non-visual effects of light [21,22]. Melanopsin retinal ganglion cells transmit signals to the suprachiasmatic nucleus (SCN), which uses this light information to regulate melatonin secretion from the pineal gland. In the presence of light, the neurons of the SCN act as the major pacemaker and entrain peripheral clocks to the external light–dark cycle [23]. The effectiveness of light exposure depends on factors such as spectrum (white vs. blue emphasis), intensity, duration, and timing relative to circadian rhythms [21,24,25]. Sunlight, rich in both white and blue light, is particularly effective due to its high illuminance that ranges from 32,000 to 100,000 lux on an average day [26]. Morning light exposure is most impactful, promoting earlier sleep [27], alertness [28], and mood [29]. Humans are most sensitive to light at night, with exposure aligning the sleep–wake cycle by suppressing melatonin production and shifting circadian rhythms [21]. Morning light exposure has been found to advance circadian rhythms [27,30,31], leading to earlier bedtimes and enhanced alertness and mood [29,32]. In contrast, evening light exposure delays circadian rhythms, resulting in a later bedtime [21,22]. Alertness, closely linked to circadian rhythms and core body temperature [33], is modulated by both circadian (timing of sleep) and sleep homeostatic processes (increased intensity of sleep with increased prior wakefulness) [34,35]. Disruption to either process diminishes alertness, impacting mood and exacerbating sleep–wake cycle disruptions [29].

In conclusion, this narrative systematic review aims to comprehensively evaluate the impact of morning short wavelength lighting (blue) and broad spectrum or blue-enriched bright white light exposure on sleep–wake rhythms, sleep disturbances, mood, and fatigue in TBI, building upon the existing literature to provide valuable insights into potential interventions for this challenging condition.

## 2. Materials and Methods

This systematic review was conducted according to the recommendations outlined in the Preferred Reporting Items for Systematic Reviews and Meta-Analyses (PRISMA) statement [36].

### 2.1. Search Strategy

An initial search was conducted on 19 September 2022 and updated monthly by search alerts until January 2024 using the following electronic databases: Medline, PsychInfo, Cinahl, Embase, and Scopus. The search strategy employed the following keywords: “Morning* light*” OR “Blue light*” OR “Monochromatic light*” OR “Bright light*” OR “Lamp light*” OR “Artificial light*” OR “Morning light exposure*” OR phototherap*” AND “Physiological change*” OR hormone* OR sleep* OR circadian* OR “Peripheral clock*” AND “health*” OR “wellbeing” OR “anxiety” OR “depression” OR “sleepiness*” OR “happiness” OR “attention” OR “wakefulness” OR “alertness*” OR “quality of life”. Covidence (Veritas Health Innovations, Melbourne, VIC, Australia) was used during the screening process of this systematic review. Two reviewers (C.M.C. and D.H.) independently screened title/abstracts for full-text inclusion and conducted the full-text screening. Manual searches of the reference lists of the included articles were performed to identify additional articles, in addition to articles from Citation Alerts, up to January 2024. Discrepancies were resolved via discussion at each stage.

### 2.2. Eligibility Criteria

Articles were eligible for inclusion if they met the following criteria: (1) study with human participants, simultaneous morning, and light exposure; (2) participants in the study had a TBI; and (3) intervention involved shorter wavelength lighting in the visible (blue) range or broad-spectrum bright white light; and (4) studies had an outcome (sleep, circadian, mood, fatigue). Articles were ineligible for inclusion if they (1) included babies or animals, (2) were conference abstracts or case studies, and/or (3) focused on light source (e.g., heat, chemical reactions), light beam, light microscopy, light pollution (inappropriate or excessive artificial light at night), and light weight (any light topic linked to physics).

### 2.3. Outcomes and Data Extraction

The outcomes for this review include phase shifts in sleep–wake rhythm, sleep variables, mood, and fatigue following morning light exposure. From the included studies, the following data were extracted: author names, year of publication, study design, participant characteristics (i.e., age, sex, and history of TBI and severity), type of light device, wavelength and intensity intervention, wavelength and intensity control, exposure prescription (e.g., timing, duration, length of exposure), sleep and circadian rhythm parameters, and mood and/or fatigue. All data extraction was completed by one author (C.M.C.).

### 2.4. Risk of Bias

The studies were assessed for risk of bias using the Joanna Briggs Institute (JBI) critical appraisal tools for randomized controlled trials (RCT) and quasi-experimental studies (experimental studies without random allocation) [37]. Two reviewers completed the risk of bias using a standardized Excel spreadsheet with any discrepancies resolved by consensus. These tools evaluated the possibility of bias in the design, conduct, and analysis of each study. Possible answers to questions were yes, no, unclear, and not applicable. An answer of “no” for any item meant the overall risk of bias was not considered low.

## 3. Results

### 3.1. Study and Participant Characteristics

**Figure 1:**
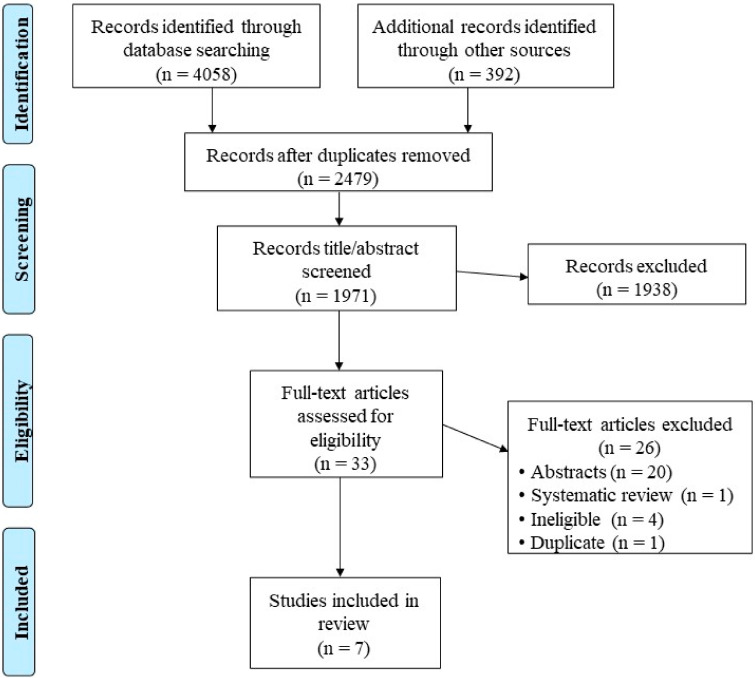
Flow chart of study retrieval process.

A flowchart detailing the study retrieval process is provided in Figure 1. Of the 4058 retrieved records, 7 studies, comprising 6 randomized controlled trials (RCTs) [12,13,14,17,38,39] and 1 quasi-experiment [16], met the inclusion criteria. Each study utilized artificial light with shorter wavelength lighting in the visible (blue) range or broad-spectrum or blue-enriched bright white light as the intervention and longer wavelength lightings of amber (530–578 nm) or red (440–480 nm), or no light as the control comparator, with one study employing two control comparators, including “no light” [14]. The sources of artificial light were administered using the GoLITE Blu^®^ (Phillips) [13,14,17,38], Litebook^®^ device [39], Light box (Aurora LightPad Mini™) [16], and Luminette^®^ Light device [12]. Light exposure duration varied, with five studies utilizing 30 min [12,13,17,38,39], one study using 45 min [14], and one study applying 60 min of light exposure [16]. All light exposure sessions occurred in the morning within 2 h of awakening or before 1100 h. The duration of light exposure ranged up to 6 weeks, with one study lasting for 10 days [39], three studies for 4 weeks [12,14,16], and three studies for 6 weeks [13,17,38]. A total of 309 participants (195 males and 114 females) aged between 21 and 53 years were included in this review. Table 1 summarizes the study characteristics.

### 3.2. Sleep–Wake Timing and Sleep Variables

The impact of morning light exposure on sleep exhibited variability across studies, with some reporting benefits while others showed no change (Table 2). This inconsistency suggests potential influences of individual characteristics, possibly including TBI severity, on intervention effectiveness. To explore this, TBI severity was categorized as mild, moderate, and severe based on the VA/DoD practice guidelines [13,16,17,38] and the Glasgow Coma Scale in two studies [12,13,17,38,39], and in one study using other methods [14,16]. Our analysis revealed that studies focusing on mild TBI generally reported more positive outcomes regarding sleep improvement compared to those investigating moderate or severe TBI.

### 3.3. Mild TBI (mTBI)

Both reported significant phase advances, with mid-sleep time shifting earlier by 30–60 min on average (Table 2). Interestingly, Killgore et al. [17] observed earlier sleep onset and offset without changes in total sleep time (TST) following morning exposure of short-wavelength lighting (blue) at 469 nm, whereas Elliot et al. [16] found a notable increase in TST (47 min on average, 21.2 ± 27.5%) alongside earlier bedtime and longer time in bed following morning broad-spectrum bright white light exposure, but no significant change was found in wake time. No significant differences were observed in other sleep variables, including sleep efficiency (SE), wake after sleep onset, number of awakenings, and activity level. For Elliot et al. [16], the improved TST corresponded with a decrease in Insomnia Severity Index score, shifting the category from moderate to mild, with no change in the sleep hygiene index. While investigating the effects of light therapy on sleep in participants with mTBI, Raikes et al. [13] found short wavelength lighting (blue) at ~480 nm in the morning did not significantly change wake after sleep onset (WASO), but self-reported SE was worse in the group exposed to short wavelength lighting (blue) at ~480 nm compared to the control group exposed to longer wavelength lighting (amber) at ~530 nm.

### 3.4. Moderate–Severe and Severe TBI (m-sTBI and sTBI)

Two studies using subjective sleep measures like the Pittsburgh Sleep Quality Index (PSQI) did not report significant changes in sleep with blue-enriched bright white light (468 nm) [12] and shorter wavelength lighting (blue) at 465 nm therapy [14], suggesting alternative approaches may be needed for these populations.

### 3.5. Sleepiness, Mood, and Fatigue

Six out of the seven studies examined the influence of light exposure on sleepiness [12,13,14,17,38,39]. Three studies involving participants with mTBI [13,17,38] and another with sTBI [14] reported a significant decrease in sleepiness. The remaining two studies showed no significant changes in sleepiness [12,39]. Two studies using the MSLT found adults with mTBI had significantly delayed daytime sleep onset after exposure to morning short wavelength lighting (blue) at 469 nm, suggesting reduced daytime sleepiness [17,38]. Interestingly, all four studies showing improved sleepiness used short wave-length lighting range (blue) between 465 and 480 nm [13,14,17,38], while the two with no significant effect used blue-enriched bright white light at 468 nm and 440-480 nm, respectively [12,39]. Regarding the two studies that examined mood, only one study by Elliot et al. [16] showed improvement in mood with broad-spectrum bright white light exposure in veterans with mTBI. Fatigue reduction was significant only in studies involving participants with sTBI [12,14].

### 3.6. Risk of Bias

The study quality evaluation, based on the Joanna Briggs Institute (JBI) critical appraisal tools, is presented in Table 3. Among the six studies that were RCTs, three exhibited excellent study quality with a low risk of bias [13,17,39], while one study had a high risk of bias due to lack of blinding of participants [12]. One study did not blind participants, deliverers of the intervention, and assessors to treatment assignment [14], and one study had several appraisal criteria marked as unclear [38]. The quasi-experimental study failed to blind participants to their treatment assignment [16].

## 4. Discussion

Our review reveals diverse effects of shorter wavelength lighting in the visible (blue) range between 465 and 480 nm or broad-spectrum/blue-enriched bright white light at a range of 440–480 nm on sleep, mood, and fatigue, depending on TBI severity and specific outcome measures. Notably, reporting the size and location of TBI in the reviewed papers proved challenging. Not all patients required medical diagnostic imaging (MDI) [41], and MDI, such as conventional brain magnetic resonance imaging (MRI), is often impractical for mTBI due to the diffuse nature of the injury and the common lack of specific findings [42]. Furthermore, the timing of injuries ranged widely, from subacute to chronic stages, spanning several years post-TBI. Such heterogeneity means that studies cannot be accurately compared. Therefore, we focused on the severity of TBI, a common denominator for injuries. Consequently, we made comparisons of light exposure outcomes according to the severity of TBI based on the VA/DoD practice guidelines and Glasgow Coma Scale scores. Morning light exposure phase advanced sleep–wake timing by 30–60 min on average in two studies [16,17], no change in WASO, decreased Insomnia Severity Index score, and improved mood [16]. Additionally, it increased or left unchanged TST in participants with mTBI. Four of six studies found a significant decrease in sleepiness [13,14,17,38]. Blue-enriched and bright white light at 468 nm and short wavelength lighting (blue) at 465 nm reduced fatigue in the severe group [12,14]. However, some mTBI studies did not assess fatigue as an outcome measure.

Morning light exposure consistently advanced mid-sleep time, sleep onset, and offset in mTBI [16,17], aligning with prior findings in healthy adults [43], patients with seasonal affective disorder [44], and those with delayed sleep phase syndrome [45]. However, not all studies observed increased TST alongside phase advances, likely reflecting individual differences in sleep onset latency and awake time during the sleep period. Phase advances represent a shift in the timing of the body clock, as evidenced by the advanced evening rise in plasma melatonin (or dim light melatonin onset, DLMO) and early timing of the core body temperature minimum. While DLMO remains the gold standard, alternative assessments like the standard deviation of the midpoint of sleep offer practical options for a wider research scope. Morning short wavelength (blue) range and broad-spectrum bright white light may be applied favorably to TBI patients with delayed sleep timing. Additionally, future studies should investigate the effectiveness of shorter wavelength lighting (blue) and broad-spectrum or blue-enriched bright white light in advancing sleep in m-sTBI individuals.

While morning short wavelength lighting (blue) range and blue-enriched bright white light exposure did not directly enhance sleep quality in m-sTBI, its benefits in mTBI suggest other avenues for sleep management in recovery. This discrepancy could be due to more extensive brain damage that weakens the pathways by which light influences sleep–wake cycles. The improvements in cognitive function and brain structure hint at light’s potential to stimulate neuroplasticity and neurogenesis, contributing to neural repair. Furthermore, optimized sleep, potentially enhanced by tailored sleep hygiene practices alongside shorter wavelength lighting in the visible (blue) range and broad spectrum or blue-enriched bright white light exposure, could play a crucial role in supporting these neural repair processes.

Beyond sleep timing, shorter wavelength lighting in the visible (blue) range and broad spectrum or blue-enriched bright white light exposure appears to hold promise for mood, sleepiness, and fatigue in TBI, with distinct effects across severity levels. Short wavelength lighting range (blue) reduced sleepiness [13,14,17,38] and fatigue [12,14] and broad-spectrum bright white light improved mood [16]. This improved mood could be partly driven by increased TST observed in Elliot’s study [16]. Since reduced sleepiness was not directly linked to improved sleep in some studies [13,14,17], light exposure might influence wakefulness through other pathways, such as neurotransmitter modulation. Neurological underpinnings may explain some of the benefits of morning exposure of short wavelength lighting in the blue range. For example, Bajaj et al. [38] provided preliminary evidence suggesting that morning short wavelength lighting (blue) at 469 nm facilitates recovery of brain structure and function following mTBI linked to reduced daytime sleepiness. Their study examined white matter water diffusion in the brain. Killgore et al. [17] further demonstrated exposure to short wavelength lighting (blue) at 469 nm showed stronger connections between a part of the brain called the thalamus and other areas involved in attention, planning, and language (frontal and parietal cortex). This was not seen with the longer wavelength lighting (amber) at 578 nm. Stronger connections between these brain areas were linked to better daytime alertness, and executive functioning. Other studies have shown patients with mTBI decreased functional connectivity between the thalamus and cortical regions a pattern which was reversed by 6 weeks of daily morning short wavelength lighting (blue) at 469 nm [17]. Interestingly, shorter wavelength lighting (blue) and blue-enriched bright white light exposure did not produce a change in sleep outcomes in both the m-sTBI [39] and severe TBI [12,14] groups, suggesting another possible factor at play in severe TBI fatigue, such as pain or cognitive load. In contrast, three studies with mild TBI demonstrated positive sleep effects and circadian phase shifts [14,16,17]. Remarkably, short wavelength lighting (blue) at 465 nm and blue-enriched bright white light at 468 nm reduced fatigue in severe TBI groups where sleep quality reported using the PSQI was not altered. In the mild TBI studies, no fatigue outcome was reported. Therefore, the effects of shorter wavelength lighting in the visible (blue) range and broad-spectrum or blue-enriched bright white light therapy in this population on fatigue are unknown. Additionally, insufficient sleep, where actual sleep falls short of individual need, has been positively correlated with daytime sleepiness, mood, and fatigue [46]. Light’s impact on these subjective measures might be mediated by its modulation of neurotransmitters known to influence mood and alertness, even with melatonin suppression [32,47]. The current evidence calls for well-designed studies to definitively explain the complex interplay between short wavelength lighting (blue) and broad-spectrum or blue-enriched bright white light exposure, sleepiness, mood, and fatigue in TBI, particularly across severity levels and considering sleep quality variations.

For participants in the included studies, heterogeneity existed in age and injury history, which is difficult if not impossible to control for, making participant selection and recruitment challenging. Notably, natural recovery post-injury could extend up to 12 weeks, yet participants in the included studies had injury histories ranging from 3 months to 55 years. Hence, any observed improvements following light treatment are unlikely due to the natural recovery process. Another point to note concerning participants was the higher male-to-female ratio, which may be explained by males’ higher participation rates in intense, physically demanding sports or military operations [48], and high-risk labor jobs due to their greater musculature.

The use of monochromatic shorter wavelength lighting (blue) with low-intensity versus polychromatic bright white light with higher intensity did not differentiate the study findings, except for sleepiness. This finding is noteworthy since shorter wavelength lighting (blue) with a peak sensitivity at 479 nm is the dominant light detected by melanopsin receptors [49]. Nevertheless, polychromatic light can be as effective in eliciting circadian responses [25], as seen in the study by Elliott et al. [16]. The reviewed studies used a light exposure range of 30–60 min, with most studies (five out of seven) using 30 min. These exposure durations appear practical, yet a dose–response curve has not been constructed. While natural light is the ideal source, all studies used light devices for standardized light exposure to minimize external factors introduced by varying light exposure levels in natural settings. However, sleep–wake timing might influence light therapy effectiveness. Since TBI can cause sleep–wake misalignment with the natural internal clock [7], this misalignment could affect how well light exposure synchronizes the rhythm. Future studies that evaluate sleep–wake patterns, advanced versus delayed, would assist with increasing the understanding of the potential benefits of light therapy in TBI patients. One fascinating variable not considered in these studies was individual chronotype, with its distinct variations in sleep–wake timing. Future research should explore this interaction between chronotype and light therapy.

Well-designed randomized controlled trials are crucial to solidify our understanding of the potential offered by short wavelength lighting (blue) range in TBI management. These studies should employ standardized protocols for light exposure, ensuring consistency in measuring key outcomes like sleep quality and quantity, sleep regularity, phase shifts, sleepiness, alertness, mood, fatigue, and chronotype. Optimal light therapy likely involves 30 min daily sessions for 4–6 weeks within two hours of awakening. Ideally, outcomes should be assessed at baseline, 3 weeks, 6 weeks, and with a 6-week follow-up. While considering the natural recovery process after TBI, starting light therapy soon after acute injury also deserves investigation.

## 5. Conclusions

Despite the lack of definitive, across-the-board effects, the existing evidence presents an intriguing picture. Morning monochromatic shorter wavelength lighting (blue) and polychromatic bright white light exposure appears to hold promise for phase advancing sleep timing, increasing total sleep time, elevating mood, and reducing sleepiness and fatigue in some TBI patients, particularly those with mild injuries. Further rigorous research, as outlined above, is crucial to unlock the full potential of this non-invasive intervention and optimize its benefits for individuals recovering from TBI.

## Figures and Tables

**Table 1:** Study characteristics.

Authors	Study Design/ Participants	History of TBI and Severity	Type	Wavelength and Intensity (Intervention)	Wavelength and Intensity (Control)	Timing/Duration/Length of Exposure
Bajaj et al. [38]	RCT (13 m, 15 f) Blue light (*n* = 14): 21.8 ± 4.4 y, 8 females); comparator placebo group (*n* = 14), mean age = 21.2 ± 3.1 y	Injury due to sports or vehicular/household accidents (≤12 mth). Mild TBI defined by VA/DoD practice guidelines.	GoLITE Blu^®^, Philips Electronics	Blue light 469 nm, 214 lux	Amber light, 578 nm, 188 lux.	Within 2 h of awakening, but before 11:00 a.m., homebased. 30 min, 6 weeks.
Bell et al. [39]	RCT (89 m, 42 f) BWL (*n* = 65); control red light (*n* = 66)	Injury caused by an external mechanical force (≤90 days). Moderate-to-severe TBI defined by Glasgow Coma Scale of 3–12.	Litebook^®^ device	BWL 440–480 nm, 1260 lux	Red light: no light emitted between 440 and 480 nm, <450 lux.	Between 7:30–9:30 a.m. during acute rehabilitation hospitalization. 30 min daily, 10 days.
Elliot et al. [16]	Single-arm, open-label pre–post-intervention (30 m, 3 f); 53 ± 18 y (*n* = 33)	3–55 y post-injury (military combat blast, blunt force, fall, sports, unknown). Mild TBI defined by VA/DoD practice guidelines.	Light box (LightPad Mini, Aurora Light Solutions Inc)	BWL (555 nm), 10,000 lux	No control group.	Morning for 60 min, 4 weeks.
Killgore et al. [17]	RCT (15 m, 17 f) Blue light (*n* = 16): 23.2 ± 7.1 y; amber light control (*n* = 16): 23.3 ± 7.4 y	Injury ≤ 18 mth (external force (e.g., head impact, blast wave)). Mild TBI defined by VA/DoD practice guidelines.	GoLITE Blu^®^, Philips Electronics	Blue light 469 nm, 214 lux	Amber light 578 nm, 188 lux.	Within two hours of awakening, but no later than 11:00 a.m. 30 min, 6 weeks.
Quera Salva et al. [12]	RCT (11 m, 9 f) BWL (10): 34.2 ± 10.7 y; control (*n* = 10): 39.0 ± 9.8 y	Cause of injury not reported (≤6 mth). Severe TBI, initial Glasgow Coma Scale score 8 or less.	Light device (Luminette Lucimed Belgium)	BWL at 468 nm, 392.2 μW/cm^2^ of one intensity of 1703 lux	No light exposure.	At awakening. 30 min, 4 weeks
Raikes et al. [13]	RCT (13 m, 22 f) Blue light (*n* = 17): 25.5 ± 8.7 y, *n* = 17; amber light (*n* = 18): 26.2 ± 7.6 y	Non-blast mTBI (≤18 mth). Mild TBI defined by VA/DoD practice guidelines.	GoLITE Blu^®^, Philips Electronic	Blue light ∼480 nm	Amber light ~530 nm.	Within 2 h of waking each morning between 8:00–10:00 a.m. 30 min, 6 weeks at home.
Sinclair et al. [14]	RCT (24 m, 6 f) Blue light (*n* = 10): 47.2 ± 13.7 y, yellow light (*n* = 10): 36.2 ± 13 y; control (*n* = 10): 42.5 ± 12.9 y, (no treatment)	TBI at least 3 mth earlier (motor vehicle injury, falls) Severe TBI based on medical records.	GoLITE Blu^®^, Philips Apollo Health	Blue light: 465 nm, 84.8 μW/cm^2^, 39.5 lux	Yellow GoLite: 574 nm, 18.5 μW/cm^2^, 68 lux.	Morning 2 h after waking, homebased. 45 min each morning, 4 weeks.

RCT = randomized controlled trial; BWL = bright white light; min = minutes; mth = months; TBI = traumatic brain injury; m = males; f = females; VA/DoD practice guidelines = Management of Concussion/mTBI Working Group, 2009 [40].

**Table 2:** Study results for the effects of morning light exposure on sleep, mood, and fatigue.

Study	Glasgow Coma Scale	TBI Classification	Circadian Phase Shift	Sleep Variables	Insomnia Severity Index	Sleepiness	Alertness/ Mood	Fatigue
Bajaj et al. [38]	Other ^1^	Mild	NM	NR	NM	Reduced sleepiness, (↑ MSLT)	NM	NM
Bell et al. [39]	3–12	Moderate–severe	NM	↔ variables	NM	↔ KSS	↔ mood	↔ fatigue
Elliot et al. [16]	Other ^1^	Mild	Phase advance of bedtime and mid-sleep time	↑ TIB, ↑ TST, ↔ in other variables	Improved (from moderate to mild)	NM	↑ mood	NM
Killgore et al. [17]	Other ^1^	Mild	Phase advance (sleep onset and offset, mid-sleep time)	↔ TST	NM	Reduced sleepiness (↓ ESS, ↑ MSLT)	NM	NM
Quera Salva et al. [12]	≤8	Severe	NM	↔ PSQI	NM	↔ ESS	NM	↓ fatigue
Raikes et al. [13]	Other ^1^	Mild	NM	↔ WASO ↔ SL	NM	↓ ESS	NM	NM
Sinclair et al. [14]	Other ^2^	Severe	NM	↔ PSQI	NM	↓ ESS	NM	↓ fatigue

↓ = decrease; ↑ = increase; ↔ = non-significant change; ESS = Epworth Sleepiness Scale; KSS = Karolinska Sleepiness Scale; MSLT = multiple sleep latency tests; NM = not measured; NR = not reported; PSQI = Pittsburgh Sleep Quality Index; SL = sleep latency; TBI = traumatic brain injury; TIB = time in bed; TST = total sleep time; WASO = wake after sleep onset. Other ^1^—VA/DoD practice guidelines 2009 [40]. Other ^2^—clinical interview and medical records.

**Table 3:** Quality of studies.

RCT	q1	q2	q3	q4	q5	q6	q7	q8	q9	q10	q11	q12	q13	q14
Bajaj et al. [38]	Yes	Unclear	Yes	Unclear	Unclear	Unclear	Yes	Yes	Yes	Yes	Yes	Yes	Yes	1
Bell et al. [39]	Yes	Yes	Yes	Yes	Yes	Yes	Yes	Yes	Yes	Yes	Yes	Yes	Yes	1
Killgore et al. [17]	Yes	Yes	Yes	Yes	Yes	Yes	Yes	Yes	Yes	Yes	Yes	Yes	Yes	1
Quera Salva et al. [12]	Yes	Yes	Yes	No	Yes	Yes	Yes	Yes	Yes	Yes	Yes	Yes	Yes	1
Raikes et al. [13]	Yes	Yes	Yes	Yes	Yes	Yes	Yes	Yes	Yes	Yes	Yes	Yes	Yes	1
Sinclair et al. [14]	Yes	Yes	Yes	No	No	No	Yes	Yes	Yes	Yes	Yes	Yes	Yes	1
**Quasi**	q1	q2	q3	q4	q5	q6	q7	q8	q9	q10				
Elliot et al. [16]	Yes	Yes	Yes	No	Yes	Yes	Yes	Yes	Yes	1				

Table 3 description: JBI critical appraisal tools for RCT [37]. q1. Was true randomization used for assignment of participants to treatment groups? q2. Was allocation to groups concealed? q3. Were treatment groups similar at the baseline? q4. Were participants blind to treatment assignment? q5. Were those delivering treatment blind to treatment assignment? q6. Were outcomes assessors blind to treatment assignment? q7. Were treatment groups treated identically other than the intervention of interest? q8. Was follow-up complete, and if not, were differences between groups in terms of their follow up adequately described and analyzed? q9. Were participants analyzed in the groups to which they were randomized? q10. Were outcomes measured in the same way for treatment groups? q11. Were outcomes measured in a reliable way? q12. Was appropriate statistical analysis used? q13. Was the trial design appropriate for the topic, and any deviations from the standard RCT design accounted for in the conduct and analysis? q14. Overall appraisal: include: 1; exclude: 2; seek further info: 3. JBI Quasi-experimental studies (experimental studies without random allocation) [37]. q1. Is it clear in the study what is the “cause” and what is the “effect” (i.e., there is no confusion about which variable comes first)? q2. Were the participants included in any comparisons similar? q3. Were the participants included in any comparisons receiving similar treatment/care, other than the exposure or intervention of interest? q4. Was there a control group? q5. Were there multiple measurements of the outcome both before and after the intervention/exposure? q6. Was follow-up complete, and if not, were differences between groups in terms of their follow-up adequately described and analyzed? q7. Were the outcomes of participants included in any comparisons measured in the same way? q8. Were outcomes measured in a reliable way? q9. Was appropriate statistical analysis used? q10 Overall appraisal: include: 1; exclude: 2; seek further info: 3.

## Data Availability

No new data were created or analyzed in this study. Data sharing is not applicable to this article.
